# Seizure detection using hierarchical temporal trend integration and self-supervised learning

**DOI:** 10.1038/s41598-026-63727-1

**Published:** 2026-08-17

**Authors:** Namitha A R, Siddartha B K

**Affiliations:** 1 Department of Computer Science and Engineering, BGS Institute of Technology -Adhichunchanagiri University, Belluru, Karnataka India; 2 Department of Information Science and Engineering, BGS Institute of Technology -Adhichunchanagiri University, Belluru, Karnataka India

**Keywords:** Epileptic seizures, Temporal sequence consistency learning (TSCLM), Federated temporal learning (FTL), Hierarchical temporal trend integration modules (HTTIMs), Temporal self supervision head (TSSH), Computational biology and bioinformatics, Mathematics and computing, Neurology, Neuroscience

## Abstract

Early, reliable detection of epileptic seizures from electroencephalography (EEG) remains challenging due to label scarcity, inter patient variability, and the non stationary nature of clinical recordings. This work introduces Federated Temporal Learning (FTL), an annotation efficient framework that couples a supervised seizure classifier with a self supervised pretext task, Temporal Sequence Consistency Learning (TSCLM), over a shared backbone. The backbone is built from Hierarchical Temporal Trend Integration Modules (HTTIMs) that extract and top down mix multi scale long and short term trends; a Temporal Self Supervision Head (TSSH) learns order relations between subsequences to inject temporal positional awareness without manual labels. Training optimizes a joint objective with a mixing coefficient δ and split ratio τ; at inference the self supervised head is discarded, yielding a purely supervised predictor. We theoretically analyze the algorithmic stability of our algorithm, and indeed, provide a bound on the gap between the empirical and the expected risk under the semi supervised objective, thereby clarifying the interplay between more labeled data and well conditioned optimization. From an empirical perspective, it also performs seamlessly at state of the art (SoA) on two datasets: TUSZ v2.0.1 (accuracy 99.16%, precision 99.19%, recall 99.65% and F1 macro 97.17%), Zenodo NICU EEG (accuracy 95.61%, precision 96.78%, recall 94.67% and F1 macro 96.87%). The results suggest that order aware self supervision in conjunction with hierarchal trend integration can be a viable solution to the label efficient and clinically reliable, EEG seizure detection task. The proposed approach has the advantage of explicitly modelling multi-scale temporal patterns by adopting a top-down strategy, using Hierarchical Temporal Trend Integration Modules (HTTIM), which is not used in existing approaches. In addition, Temporal Sequence Consistency Learning (TSCLM) is designed as an order-aware self-supervised task to capture temporal relationships without labels. The integration of these components within the Federated Temporal Learning (FTL) framework enables improved performance under limited annotated data.

## Introduction

Reducing mortality through prevention, timely treatment, and the promotion of mental health and well-being are central aims of the Sustainable Development Goals. Early detection and accurate classification of epilepsy can substantially reduce epilepsy-related mortality and alleviate the stigma and discrimination experienced by patients and their families. Epilepsy is a non-communicable neurological disorder that contributes significantly to premature death, particularly in low- and middle-income countries, where children and the elderly are especially vulnerable. Historical records indicate that epilepsy was first documented in ancient inscriptions more than 4000 years ago^1^. A seizure results from abnormal, excessive, or synchronous neuronal activity in the brain and may or may not be accompanied by awareness of the event. In some cases, seizures lead to involuntary movements, while in others they manifest without any observable motor activity. Although not all abnormal brain activity qualifies as a seizure, the occurrence of seizures reflects a malfunction of the brain. Given that the brain is responsible for functions such as planning, voluntary motor control, sensory processing, perception, awareness, personality, emotional regulation, memory, and visual reasoning, any disruption can impair one or multiple functions. Such dysfunctions may lead to severe morbidity, increased mortality, and long-term adverse effects on mental health^2,3^.

Epilepsy is a major public health problem, impacting ~ 65 million people worldwide, and ~ 2 million new cases diagnosed annually^4^. Elevated morbidity and mortality have been correlated with seizures, in part because of the loss of consciousness that occurs during the seizure and the possibility of injuries during the event, and the fact that if a seizure is prolonged it may lead to irreversible brain damage^5^. Also, seizures that happen while the child is sleeping may be life-threatening (sudden unexpected death^6^. Hence, an automatic detection and forecasting of epileptic seizures is a critical clinical and research problem that is in an urgent need^7^. EEG has long been the main tool of epileptologists for detection and characterization of seizures leading to source location and analysis of seizure propagation between brain regions^8^. As mentioned before, the signals acquired with the EEG are often complex and not stationary, not allowing an expensive, subjective visual examination and, therefore, not suitable for immediate clinical application. Therefore, the demand for automated techniques which detect seizures is increasing^9^.

Many studies have been devoted to this challenge over the years, employing one or more machine learning (ML) technique(s)^10^. Support vector machines (SVM), random forest and decision trees are among the classical ML classifiers which have proven to be effective in the detection of epileptic seizures^11^. However, they are highly reliant on the availability of adequate amount of labeled data for training and validation. In recent years, Deep Learning (DL) has become a significant paradigm able to learn deeper and more discriminative patterns from massive amounts of data thanks to a multi-layer hierarchical architecture. Recently, its outstanding performance in these real applications brought its success of detection of epilepsy also^12^. Specifically, Convolutional Neural Networks (CNNs) and Recurrent Neural Networks (RNNs), such as Long Short-Term Memory (LSTM) networks have exhibited a high performing ability in EEG classification problems^13^. Recently, attention mechanisms have been added to these models to strengthen them with the most informative temporal and spatial EEG features^14–16^.

Although several improvements have been made with classical machine learning (ML) methods and advances in deep learning (DL) in recent years, current automated seizure detection (ASD) systems still have some limitations: they require huge amounts of labelled EEG information, are not easily extented to the highly non-stationary and non-stationary nature of EEG data across patients and recording contexts and might not generalize well in a real clinical context. To this end, there is a need for an automated seizure detection and classification framework that can reliably classify seizures from non-seizure activity (and, in the case of need, different seizure types) from EEG signals, have less reliance on the time-intensive manual annotation process and provide reliable and clinically meaningful assistance for early diagnosis and intervention.

The novelty of the work is the fact that it integrates order-aware, self-supervised learning with hierarchical temporal trend modelling in a unified framework. In addition to long and short-term temporal dependence, explicit, HTTIM also explicitly captures short-term temporal dependency, and TSCLM improves temporal representation learning without labeled data. This combination improves generalization and distinguishes the proposed method from existing EEG-based approaches.

The rest of this paper is organized as follows. The related work is discussed in Sect.  2. The proposed methodology is given in Sect.  3. The experimental setup is described in Sect.  4. Results are presented in Sect.  5. Lastly, Sect.  6 is followed by the conclusion.

### Motivation and contribution

Epilepsy continues to be a serious health threat worldwide and carries long-term psychological and social repercussions and the risk of injury and sudden death in sleep due to unanticipated and recurrent seizures. Access to specialist care is limited and vulnerable groups are at greater risk of harm in low and middle income countries in particular, thus undermining the burden. Prompt, accurate identification of seizures is thus an essential step in prompt intervention, death prevention and better quality of life for patients.Continuous visual expert inspection is time consuming, subjective and impractical for large-scale or real-time monitoring of epileptic activity; however, electroencephalography (EEG) is the major method used to characterise activity. While machine learning and deep learning algorithms have contributed to maintaining the high detection accuracy, many of the existing methods continue to depend on large amounts of labeled data, have been a challenge to adapt for inter-patient variations, and are difficult to apply across different recording settings. These limitations motivate the development of automated, robust, and label-efficient seizure detection frameworks that can fully exploit EEG signals for reliable clinical decision support.

- Unified semi-/self-supervised training (FTL). We introduce *Federated Temporal Learning* that jointly optimizes a supervised seizure classifier and a self-supervised pretext task on a shared backbone,
- Hierarchical Temporal Trend Integration (HTTIM). We design HTTIM blocks that top-down fuse long- and short-term trends using multi-kernel convolutions and learnable sequential mixing, expanding the receptive field while preserving short-horizon detail.
- Order-aware self-supervision (TSCLM/TSSH). The *Temporal Sequence Consistency Learning Module* forms intra-/inter-sequence segment pairs under a split ratio τ\tauτ and trains a *Temporal Self-Supervision Head* to predict the correct ordering, injecting temporal positional awareness without labels.

## Related work

In^9^, present an unsupervised deep learning approach for seizure detection based on a VariationalAutoencoder (VAE), a probabilistic extension of the traditional autoencoder. The VAE is trained exclusively on seizure-free EEG recordings and learns a compact latent representation that characterizes the distribution of normal brain activity, so that deviations associated with seizures can be detected. Although the training process does not explicitly use seizure labels and is described as unsupervised, the selection of seizure-free segments still depends on expert annotation, introducing a degree of “partial supervision.” Even with this limitation, the method achieves satisfactory detection performance. In a related study^10^, design an unsupervised seizure detection scheme using a Generative Adversarial Network (GAN) tailored for behind-the-ear EEG recordings. One-dimensional EEG signals are transformed into spectrogram images using Short-Time Fourier Transform (STFT), encoding time–frequency information in a visual form. A GAN is then trained on these spectrograms, where the generator learns to synthesize realistic images of normal EEG and the discriminator distinguishes genuine images from generated ones. Training is carried out only on seizure-free data identified by experts, and the results on a proprietary dataset are encouraging.Clustering-based and hybrid signal processing strategies have also been widely explored. K-means clustering, a classical unsupervised algorithm that groups data according to proximity to cluster centroids, is used in^11^ in combination with ensemble empirical mode decomposition (EEMD) and marginal spectrum analysis to detect epileptic seizures from EEG signals. In^12^, the authors introduce a feature extraction pipeline based on masking and check-in mechanisms to obtain discriminative attributes from EEG, followed by K-means and K-Nearest Neighbors (KNN) classifiers to distinguish normal from abnormal activity. Gaussian Mixture Models (GMMs) extend the idea of K-means by modeling each cluster as a separate Gaussian distribution and assigning data points to clusters in a probabilistic (soft) manner, which leads to more flexible and often more accurate clustering compared with the hard assignments of K-means. Other activities involve incremental learning, temporal dynamics, and spiking neural computation. In^13^ an incremental learning strategy for seizure detection is developed, called SAR-LTS. By recognizing that short-term neural activity is similar, SAR-LTS takes advantage of this similarity, and uses a similarity-aware mechanism and local temporal sampling to construct an experience pool of representative EEG samples. The samples are replayed randomly over the year but segmented to enhance retention of past knowledge and to prevent catastrophic forgetting. In^14^ an intelligent seizure detection is built using two commonly used imbalanced EEG data sets. The signals of the Electroencephalogram are first transformed through the Slantlet Transform (SLT) to extract multiresolution features in the time–frequency domain, with better localization. The extracted features are, then, fed into a spiking neural network (SNN) and a hybrid SLTSNN network for epileptic seizure analysis is formed.Features extracted are then fed into a spiking neural network (SNN) to form a hybrid SLTSNN network used for epileptic seizure analysis. In recent work, several approaches tackle the issues of interpretability and generalization across patients. In^15^ a new model called the Variational Sparse Autoencoder (VSAE) is proposed, which is an abstraction of Sparse Autoencoders and Variational Autoencoders to create compact, informative and sparse representations. These features are then classified using a Random Forest (RF) classifier. To enable transparency, Explainable AI (XAI) methods are included: LIME is used for local and instance-level explanations, while SHAP is applied to generate global explanations highlighting the most important EEG features involved in seizures. In^16^, a cross-patient seizure detection setting is investigated. Pre-processing and Data generation technique is described to transform EEG signals as suitable input for Convolutional Neural Network (CNN) and the architecture and performance of CNN in addressing Inter-subject variability in detail. Recent research leverages contrastive learning, simple neural networks, and brain connectivity analysis through graph models. In^17^ the authors present a pipeline for seizure prediction that achieves a high performance even in the presence of a small number of labeled examples, thus minimizing reliance on full-and complete manual annotation. In order to improve the separativeness of the features between interictal and preictal state, contrastive learning is performed to introduce the separativeness between preictal and interictal features of the recorded signals in order to improve their sensitivity to early seizure state. A dedicated data augmentation scheme was used to boost robustness and generalization which involved both wavelet-based frequency mixing and temporal masking, and window-based masking. A hierarchical contrastive loss that fuses instance-level and temporal contrastive learning further refines the representation of preictal activity, while a lightweight SE-EEGNet is optimized as a feature extractor for real-time prediction. In^18^, spatiotemporal dynamic effective connectivity networks (ST-DECNet) are introduced to characterize time-varying nonlinear effective connectivity in the brain. A gated temporal convolutional network based on neural Granger causality (GTCN-NGC) first estimates dynamic effective connectivity networks from multichannel EEG signals with automatic lag selection.

The networks are subsequently encoded to 2D graphs, which are then sent to a graph isomorphism network (GIN) processor equipped with attention mechanisms and capable of learning complex spatiotemporal structure.An approach to classify epileptic seizure with low complexity based on separable Gabor wavelets is developed in^19^. First, continuous wavelet transform (CWT) is applied to build EEG scalogram sequences to describe the time–frequency content of the EEG signals. They are represented as scalograms which are processed with a separable Gabor wavelet filter bank to obtain distinct features and the features are combined into a fixed sized feature vector. Finally, the dimensionality is reduced using Linear Discriminant Analysis (LDA) to keep the most discriminative components. Lastly, a Support Vector Machine (SVM) classifier is used to divide the segments into non-seizure and epileptic seizure. The classification performance is validated against some of the state-of-the-art algorithms for seizure detection with reduced computational requirements, and shows competitive classification accuracy. The proposed technique has three distinctions with respect to the existing approaches. First, HTTIM explicitly models multi-scale temporal features using hierarchical fusion, unlike conventional CNN and RNN methods. Second, TSCLM introduces an order-based self-supervised task that captures temporal relationships, whereas most existing methods rely on reconstruction or contrastive learning. Third, the FTL framework jointly optimizes supervised and self-supervised objectives, improving performance under limited labeled data.

The current frameworks used in detecting seizures often depend on a completely supervised learning paradigm, centrally trained model or a single temporal-level analysis. It is a common problem that these approaches need large datasets of annotations, have privacy implications and are not very adaptable for variable clinical settings.

To overcome these shortages, this study comes up with the NSS framework which combines Federated Temporal Learning, Hierarchical Temporal Trend Integration Modules, Temporal Sequence Consistency Learning, and swarm-based optimization to enhance the scalability, privacy preservation, and seizure detection performance in the face of limited labeled data.

## Proposed methodology

**Fig. 1:**
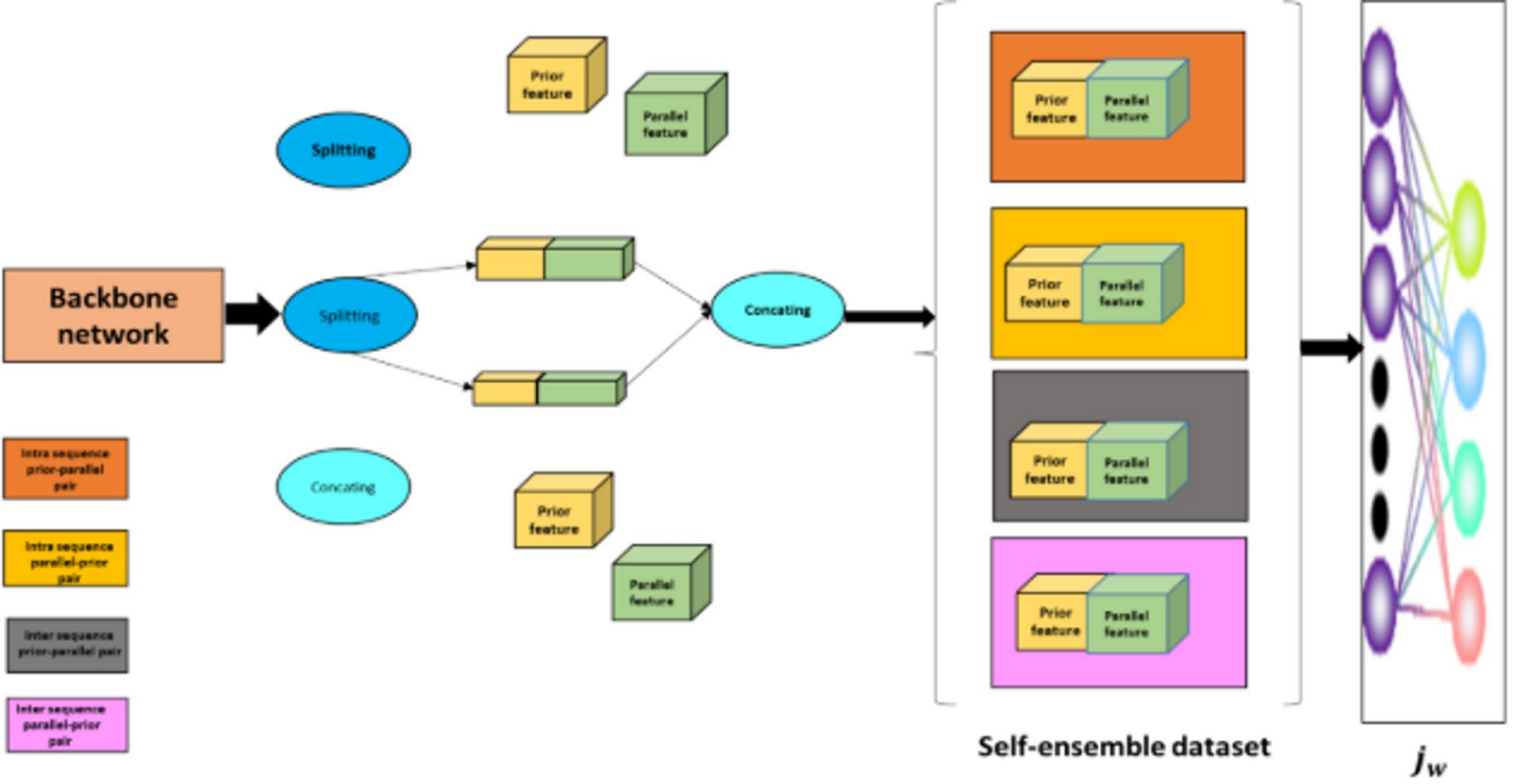
Proposed architecture.

An initial step in this section is to describe the task. Next, we initiate the self-supervised learning process within the proposed framework. Shared backbone and TSCLM task. Then, we give the description of the supervised learning of the classification task. Finally, we combine the guided and learner-guided learning to introduce the Federated Temporal Learning (FTL) framework and theoretical discussion of the uniform. Federated Temporal Learning (FTL) framework error in generalization and stability. Proposed Architecture are in Fig. 1.

### Preliminaries and problem definition

In this paper, denote lower case letters for scalars (e.g., * a*), upper case letters for vectors (e.g., * A*) and upper case boldfaced letters for matrices (e.g., * A*). A time series is a real-valued and ordered observations. We denote a time series

$$\:A=\left[{a}_{\left(\mathrm{1,1}\right)}{a}_{\left(\mathrm{2,1}\right)}{a}_{(v,1)}{a}_{\left(\mathrm{1,2}\right)}{a}_{\left(\mathrm{2,2}\right)}{a}_{(v,2)}{a}_{(1,f)}{a}_{(2,f)}{a}_{(v,f)}\right]$$.

Here * v* denotes the number of annotations and* f*denotes the number of variables. A time series is referred to as multivariate wherein * f>1* and denoted as univariate if* f=1*.

In the SSL database $$\:\alpha\:=({\alpha\:}^{n},{\alpha\:}^{w})$$ consisting of two parts, for the labelled dataset $$\:{\alpha\:}^{n}=\{\left({Z}^{\left(k\right)},{A}^{\left(k\right)}\right){\}}_{k=1}^{{p}_{n}}$$, by considering the input target pairs within time series $$\:Z\in\:{T}^{{p}_{n}*f*v}$$ and the respective one-hot encoded label $$\:A\in\:{T}^{{p}_{n}*e}$$ wherein e stands for the number of classes, the unlabelled dataset $$\:{{\alpha\:}^{w}=\{\left({Z}^{\left(k\right)}\right){\}}_{k=1}^{{p}_{w}}}^{}$$, by having only the input time series $$\:Z\in\:{T}^{{p}_{w}*f*v}$$ and the labels are not noted. However$$\:{p}_{n}$$ and $$\:{p}_{w}$$ represents the number of samples within the labelled and unlabelled datasets. By consideration $$\:{p}_{n}$$<$$\:{p}_{w}$$in the SSL field. Parallel the labelled dataset $$\:{\alpha\:}^{n}$$ is fed to shared backbone network $$\:{h}_{\theta\:}$$ following a supervised head $$\:{j}_{n}$$for a supervised task. The unlabelled dataset $$\:{\alpha\:}^{w}$$ is given to a shared backbone network $$\:{h}_{\theta\:}$$ followed by a self-supervised head $$\:{j}_{n}$$ for the supervised task .the main aim is to model the self-supervised task to perform feature extraction model on $$\:{h}_{\theta\:}$$ to understand the set of hidden representations through unseen structured data.

The self-supervised learning has been modeled as subdivided into two part networks: a shared backbone network $\:{h}_{\theta\:}$ (consisting with 9 Hierarchical Temporal Trend Integration Module (HTTIM blocks and three BN + ReLU layers of interconnections in the head network) and an autonomously operated head network $\:{j}_{w}$. The shared backbone network $\:{h}_{\theta\:}$ deploys nonlinear transform between low-level temporal features and multiscale hierarchically guided trend features. Superior head network $\:{j}_{w}$ is self-supervised seeks to find out that underlying relation of order between them. Self-sequences.

#### Temporal sequence consistency learning module (TSCLM)

By formation of the most representative data used by raw time series that can be used to predict the order relationships. In the case of temporal data, the field of receptivity may be said to be a theoretical measure defining the maximum capacity of neural network to interpret in a 1-D space. An increase in receptive field in theory increases the network ability to identify extended patterns. Conversely, a smaller receptive field helps the network to better identify shorter patterns. It is based on the previous one by applying big convolution kernels to long-term-trend are considered the features that are extracted with short-term trend features known as small convolution kernels. Although these techniques can reflect long term trend short term trend features independently, and our short-term feature vision goes far beyond that. We would like to be able to create short and long-term mixing trend feature representations of downstream tasks. Inspired by the previous block, by suggesting the HTTIM block to be extracted on multiscale hierarchically directed trend features.

By including different trend features by operating on the input tensor $\:{Z}^{(t,n)}\in\:{T}^{f*v}$ through four separate convolutional kernel sizes wherein * t*denotes * tth*residual block $\:(t=\mathrm{1,2},3)$ and n denotes nth layer in the residual block $\:(n=\mathrm{1,2},3).$ The output of the convolution operation represents $\:{Z}^{u\left|\right(t,n)}\in\:{T}^{\frac{j}{4}*v}$, $\:u=\mathrm{1,2},\mathrm{3,4}$ ,here * j* denotes channels in the hidden layer and u denotes different trend feature level. Then, these trend becomes hierarchically aligned, these features are added to the LSM one after another by guiding the trend features. The following is the LSM formula:

$$\:{Z}^{u+1|\left(d,n\right)}={Z}^{u+1|\left(t,n\right)}\left({h}_{sigmoid}\left({Z}^{u|\left(t,n\right)}{Y}^{u}\right)\right)$$

Wherein here the trainable parameters within the * uth* ($\:u=\mathrm{1,2},3$) LSM is represented by $\:{Y}^{u}$ the convolution operator is represented by and the elementwise product operator is represented by the activation function is represented by $\:{h}_{sigmoid}$ to correct the optimal learning behaviour. The process that continues until there is top-down mixing strategy is achieved.

Wherein here the trainable parameters within the * uth* ($\:u=\mathrm{1,2},3$) LSM is represented by $\:{Y}^{u}$ the convolution operator is represented by ⨂ and the elementwise product operator is represented by ⊙ the activation function is represented by $\:{h}_{sigmoid}$ to correct the optimal learning behaviour. The process that continues until there is top-down mixing strategy is achieved.

#### Temporal self-supervision head (TSSH)

The largest scale gives features of short-term trends which are dictated by the long-term trend features. Note that we discard the bottom-up mixing strategy. We prefer a top-down mixing strategy since the long-term trend characteristics make more sense. The macrolevel insights, and the short-term trend features are represented oftentimes produce noise The mixing approach is the bottom-up strategy which generates multiscale. This hierarchically steered trend features which are more unstable and prone to interference by noises. By comparisonand combining all multiscale hierarchy guided trend features to additional learning of representation.

Sequential order has a major role in data of time-series. Numerous studies have shown that temporal analysis methods are prone to the loss. temporal information which is important in high accuracy In order to increase the awareness of the position of the shared network $\:{h}_{\theta\:}$, we suggest the TSCLM pretext task, entrenching relation of order into the common backbone network. The unlabelled data * Z* is fed into a shared backbone $\:{h}_{\theta\:}$ to obtain the features as $\:Z\in\:\:{T}^{j}$. Considering a split ratio $\:\delta\:$ (0<$\:\delta\:<1),\:thesefeaturesB$ are divided into two segments wherein the front section denotes the features prior to $\:{U}^{+}{T}^{j*\delta\:}$ and the rear segment represent subsequent features. $\:{U}^{-}{T}^{j*(1-\delta\:)}$. The two segments $\:{U}^{+}$ and $\:{U}^{-}$ are randomly combined in a sequence $\:J\in\:{T}^{j}$ to build a assembling dataset as $\:{\alpha\:}^{j}=\{{J}^{\left(k\right)},a){\}}_{k=1}^{{p}_{w*4}}$. that gathering four cases: intra-sequence prior subsequent, intra-sequence subsequent-prior segment pair segment pair, inter-sequence prior-subsequent segment pair and inter-sequence preceding/succeeding segment pair.

$$\:{\sigma\:}_{scp}=-\frac{1}{\left|{F}^{j}\right|}{\sum\:}_{k=1}^{\left|{F}^{j}\right|}{A}_{k}.log\left({R}_{k}\right)$$

#### Supervised learning

**Fig. 2:**
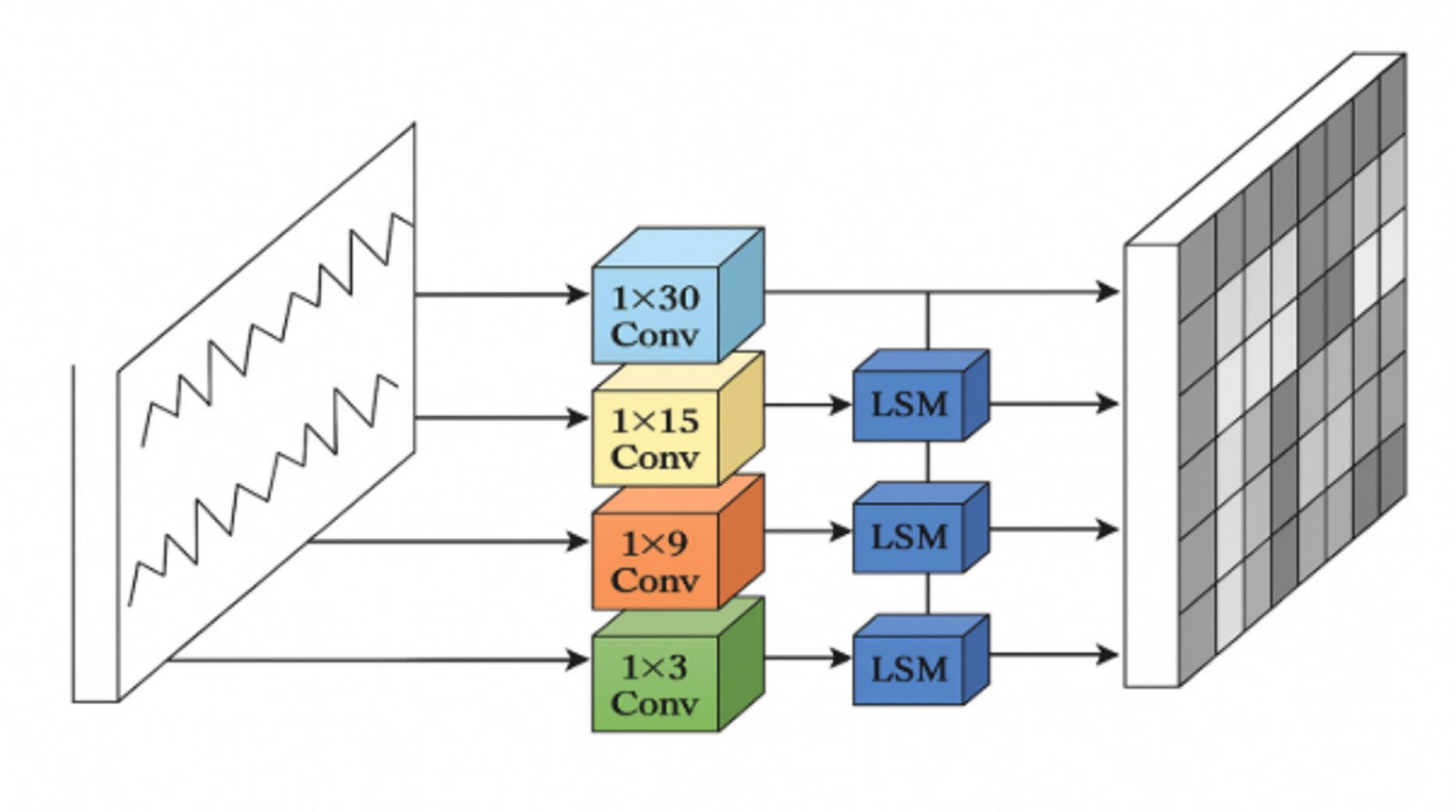
Shared backbone.

Considering the supervised head network $\:{j}_{n}$ ,by leveraging the considering multiscale hierarchically guided trend features to provide the output for probabitlity distribution over various labels. Shared backbone are in Fig. 2. The supervised training loss is defined as the cross entropy in between the probability distribution * R* over labels and the real labels * A* as given by the follow equation:

$$\:\mathrm{L}\hspace{0.17em}=\hspace{0.17em}\mathrm{L}\_\mathrm{c}\mathrm{l}\mathrm{s}\:+\:(1\hspace{0.17em}-\hspace{0.17em})\:\mathrm{L}\_\mathrm{s}\mathrm{c}\mathrm{p}$$

where L_cls denotes the supervised classification loss, L_scp represents the self-supervised sequence consistency loss, and δ is the balancing coefficient.

#### Federated temporal learning (FTL) framework

The fundamental goal of annotation-free multitask learning is to do the main classification work, but it also tries to gain something meaningful out of the auxiliary pretext tasks. The main job of classifying is called FTL, and we use the TSCLM task to make it better. During the training phase, FTL trains both the main and pretext duties at the same time. They have the same backbone that helps them find trend aspects that are directed at several scales and levels. The TSCLM task helps the backbone figure out how things are related to one other. During the testing process, FTL takes off the self-supervised head to test the sample. By employing a hard-parameter **δ** technique to control the balanced mixing ratio of both tasks during training. This helps us better balance the main and pretext tasks in our methodology. however, the equation is defined as:

$$\:{\sigma\:}_{n}=\delta\:{\sigma\:}_{cls}+(1-\delta\:){\sigma\:}_{scp}$$

**Algorithm 1:** Proposed model.

Requirement	Labelled samples (* Z,A)* and unlabelled samples Z; shared back-bone network $\:{h}_{\theta\:}$; supervised head network $\:{j}_{n}$ ;self-supervised head network $\:{j}_{w}$ ; split ratio , balanced mixing ratio $\:\delta\:$
Step 1	For * v* in [1,num_epochs] do
Step 2	For each minibatch$\:{p}_{d}$ do
Step 3	R<-$$\:{j}_{n}\left({h}_{\theta\:}\left(Z\right)\right),Z \in {T}^{{p}_{d}*f*1}$$
Step 4	B<-$$\:{h}_{\theta\:}\left(Z\right),Z \in {T}^{{p}_{d}*f*1}$$
Step 5	$\:{U}^{+},{U}^{-}$<-split (B, ***τ***),B$\: \in {T}^{{p}_{d}*j}$
Step 6	$\:{J}_{0},{A}_{0}<-concat\left({U}_{k}^{+},{U}_{k}^{-}\right),k=l$
Step 7	$\:{J}_{1},{A}_{1}<-concat\left(random\left({U}_{k}^{+}\right),{U}_{l}^{-}\right),knotequaltol$
Step 8	$\:{J}_{2},{A}_{2}<-concat\left({U}_{k}^{-},{U}_{l}^{+}\right),k=l$
Step 9	$\:{J}_{3},{A}_{3}<-concat\left(random\left({U}_{k}^{-}\right),{U}_{l}^{+}\right),knotequaltol$
Step 10	$\:J<-\{{J}_{0},\:{J}_{1},{J}_{2},{J}_{3}\}$
Step 11	$\:A<-\{{A}_{0},\:{A}_{1},{A}_{2},{A}_{3}\}$
Step 12	$\:R<-{j}_{w}\left(j\right)$
Step 13	$\:{n}_{n}$<- $\:\delta\:{\sigma\:}_{cls}$ +(1-$\:\delta\:){\sigma\:}_{scp}$ using Eqs. (2) and (3)
Step 14	End for
Step 15	End for
Step 16	Return shared backbone network $\:{h}_{\theta\:}$ and supervised head $\:{j}_{n}$

### Theoretical analysis

This section provides theoretical analysis of proposed Federated temporal learning (FTL) framework. Write and explain the algorithmic stability and generalisation of model. The analysis reformulates the objective function to assess the model’s uniform stability by introducing a revised loss function that isolates the semi-supervised time-series classification component. The study defines key assumptions δ-admissibility, strong convexity, and low-dimensional embedding assumptions to ensure the stability and convexity of the optimization problem. Based on these, the stability coefficient ξ is derived to represent how sensitive the model is to changes in training data.

The section also derives a generalization bound that links the empirical risk and expected risk, demonstrating that Federated Temporal Learning (FTL)framework. Generalization ability is influenced by both the stability of the optimization process and the classification task. As a result, the generalization gap goes down as if more labeled training examples were added, implying better generalization, thus reducing the overfitting in the system output. Finally, the theorem suggests that by increasing the number of labled samples and using more well conditioned optimization, the performance of the Federated Temporal Learning (FTL) framework is improved, thereby, making the model efficient and robust.

## Performance evaluation

A consistent increase in the accuracy of seizure detection over time is shown for successive deep learning performance evaluations. Moderate results have been obtained by early CNN and RNN based approaches, and later architectures with attention, graph learning and multi-scale feature extraction have brought high gain in performance. The proposed models showed good generalization in the accuracy, precision, recall and F1-scores on all datasets. Collectively, the findings validate the reliability of classifications involving advanced feature fusion and the use of adaptive learning strategies compared with the current state-of-the-art. The term “proposed model” is used throughout the report for consistency, and signifies the Federated Temporal Learning (FTL) framework discussed in the methodology section.

To prevent data leakage, the data was split using patient-wise splitting method to decrease the chance of learning outcomes to some extent.To avoid data leakage, data splitting was done in three sections, namely 70% training, 10% validation, and 20% testing. The EEG signals were subdivided into one-second epochs. To overcome the issue of class imbalance, all the seizure samples were used and number of samples representing non-seizure (norm) samples was randomly selected during the training phase. The model was trained with Adam optimizer and learning rate 0.001, 100 epochs and a batch size of 32. The mixing coefficient δ was set to 0.7, and the split ratio τ was set to 0.5. Ablation studies were performed by removing HTTIM, TSCLM, and the joint training mechanism to evaluate their individual contributions.

### Dataset details

#### TUSZ^19^

In this study, the EEG data set archived at Temple University Hospital (TUSZ v2.0.1) is used, which contains 675 patients’ recordings. Of these, more than 280 people had at least one seizure – totaling over 3,500 recorded seizures. The total recording time is over 5 million seconds of which the seizure portion typically accounts for 4–6% of the data set. TUSZ is one of the largest and widest available publicly available EEG repositories focused on epilepsy research. It contains a wide range of clinical information such as patient attributes (age, gender), medical history, frequency of seizures and drug details. The dataset encompasses eight types of seizures: FNSZ, GNSZ, ABSZ, CPSZ, TCSZ, TNSZ, SPSZ, and MYSZ, along with BCKG annotations denoting background activity. Because of the rarity of MYSZ events, only the other seven classes, the majority, were included in the present study.

#### Zenodo^20^

The Zenodo dataset includes multi-channel EEG recordings from 79 term neonates admitted to Neonatal Intensive Care Unit (NICU) at Helsinki University Hospital, averaged 74 min in length. Three expert clinicians rated each recording independently and on average about 460 seizures per clinician. Consensus led to 39 neonates being identified as having seizures, 22 as seizure-free, and the rest were inconclusive. All of the EEG signals were segmented in to 1-second epochs to facilitate consistent preprocessing. Non-seizure segments were temporally divided by non-overlapping windows and seizure segments were temporally divided by overlapping windows to achieve capturing the brief and rare segment of seizure. Specifically, seizure segments from the CHB-MIT and Zenodo datasets were filtered by 50% overlap, and those from the TUSZ and AUBMC datasets by 75% overlap due to the lesser number of specific seizure types. Any time period that covered a period of time during which seizures occurred was classified as seizure and the remainder as non-seizure. To overcome the inherent class imbalance of the seizure detection task, training dataset was created by keeping all of seizure segments and randomly sampling equal number of non-seizure segments. The proposed strategy improves the model’s power to learn discriminative features to robustly detect seizure from heterogeneous clinical datasets.

### State-of-art techniques

- CNN-RNN (2020)^21^: CNNs extract spatial EEG features while RNNs model temporal dynamics, capturing short- and long-term dependencies for seizure recognition.
- GASF-CNN (2021)^22^: Converts EEG time series to GASF images and applies CNNs to learn spatial correlations, improving classification accuracy.
- SincNet-Conv1D (2021)^23^: Uses parameterized sinc filters to learn frequency-selective features directly from raw EEG, boosting efficiency and interpretability.
- GGN (2022)^24^: Represents EEG as a channel–connectivity graph; graph convolutions capture spatial dependencies for robust, cross-subject detection.
- LightSeizureNet (2022)^25^: Lightweight depthwise‑separable CNN with residual shortcuts for real-time seizure detection on constrained hardware.
- EEGNet (2023)^26^: Compact CNN combining temporal filtering with depthwise spatial convolutions; effective on limited data and remains interpretable.
- Meta-GNN (2023)^27^: Meta-learning within a GNN adapts to inter‑patient variability, enhancing cross-subject generalization with few labels.
- 3D-CBAMNet (2023)^28^: 3D convolutions plus channel/spatial attention emphasize seizure‑relevant spatiotemporal regions.
- RNN (2023)^29^: Models temporal correlations in EEG but can suffer vanishing gradients compared with gated variants.
- CNN and RNNs (2024)^30^: Hybrid pipeline where CNN features are temporally contextualized by RNN layers, balancing spatial and temporal cues.
- ResBiLSTM (2024)^31^: Residual connections with BiLSTM capture bidirectional dependencies while mitigating gradient issues.
- Stacked LSTM (2024)^32^: Multi-layer LSTMs learn higher‑order temporal structure for long-record seizure detection.
- PCC-CNN (2024)^33^: Integrates Pearson-correlation features with CNNs to exploit inter‑channel synchronization changes.
- Two-Layer LSTM (2024)^34^: Stacked two-layer LSTM strengthens long‑term dependency learning and temporal coherence.
- GGN-BiGRU (2024)^35^: Graph-based spatial encoding combined with bidirectional GRUs for stronger spatiotemporal modeling.
- MultiSincNet (2024)^36^: Parallel sinc-filter paths learn multi-band spectral features, improving noise robustness.
- DistilCLIP-EEG Teacher [37] [ES]: High‑capacity contrastive/distillation model aligning multimodal EEG embeddings to supervise student networks.
- DistilCLIP-EEG Student [37][ES]: Distilled, lightweight model that retains the teacher’s discriminative power for real‑time deployment.

### Results

#### Results on TUSZ dataset

**Fig. 3:**
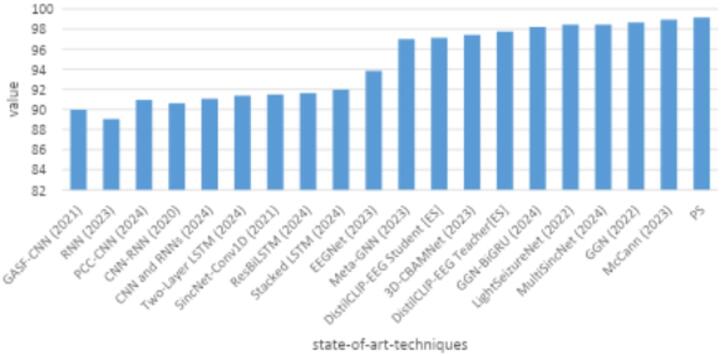
Comparison on state-of-art techniques for accuracy on TUSZ dataset.

Table 1 shows a relative improvement in accuracy (%) over time for the different models of seizures detected across the different accuracy units. A few of the previous frameworks had their accuracy as 89.04% (RNN 2023), 89.96% (GASF-CNN 2021) and 90.61% (CNN-RNN 2020), respectively. The following models achieve moderate improvements over this range (PCC-CNN in 2024 at 90.94% and CNN and RNNs in 2024 at 91.37%, as well as Two-Layer LSTM in 2024 at 91.37%). SincNet-Conv1D (2021), ResBiLSTM (2024), and Stacked LSTM (2024) further refined performance with accuracies of 91.48%, 91.63%, and 91.96%, respectively. Further, more modern versions brought radical enhancements, achieving a score of 93.84% in the case of EEGNet (2023), and 97% for Meta-GNN (2023). The accuracy for the proposed DistilCLIP-EEG Student [ES] Model and Teacher [ES] Model were 97.12% and 97.75%, respectively, which is a good generalization result. State-of-the-art architectures such as GGN-BiGRU (2024), MultiSincNet (2024), LightSeizureNet (2022), and GGN (2022) delivered near-saturated performance, ranging from 98.19% to 98.64%. McCann (2023) further advanced accuracy to 98.93%, while the proposed PS model achieved the highest accuracy of 99.16%, underscoring its superior capability in accurate seizure classification. Comparison on state-of-art techniques for accuracy on TUSZ dataset are in Fig. 3.

**Table 1:** Accuracy (%).

Methods	Accuracy (%)
CNN-RNN	90.61
GASF-CNN	89.96
SincNet-Conv1D	91.48
GGN	98.64
LightSeizureNet	98.42
RNN	89.04
EEGNet	93.84
Meta-GNN	97
McCann	98.93
PCC-CNN	90.94
CNN and RNNs	91.37
Two-Layer LSTM	91.3
ResBiLSTM	91.63
Stacked LSTM	91.96
GGN-BiGRU	98.19
MultiSincNet	98.55
DistilCLIP-EEG Student [ES]	97.12
DistilCLIP-EEG Teacher [ES]	97.75

**Fig. 4:**
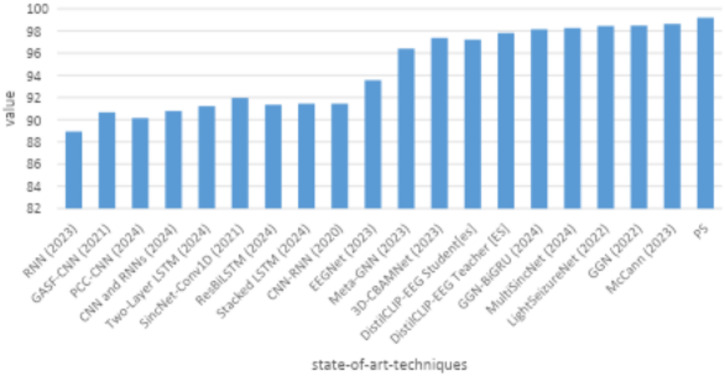
Comparison on state-of-art techniques for Precision on TUSZ dataset.

When the seizure detection models are compared based on precision (%), it can be observed that the classification accuracy of the models improves with time. The early works such as RNN (2023) and GASF-CNN (2021) had a precision of 88.93% and 90.67%, respectively; whereas CNN-RNN (2020), PCC-CNN (2024) and CNN and RNNs (2024) brought modest enhancements in the ranges of 90.15% to 90.78% precision. Two-Layer LSTM (2024), SincNet-Conv1D (2021), ResBiLSTM (2024), and Stacked LSTM (2024) were found with values of 91.22% to 91.97%. EEGNet (2023) achieved a precision of 93.56%, and the advanced models, like 3D-CBAMNet (2023) and Meta-GNN (2023), recorded a precision of 96.4% and 97.38% respectively. The proposed models Student [ES] and Teacher [ES] achieved highly accurate precision with values of 97.21% and 97.81% respectively, showing the high accuracy of the models with respect to keeping precise across seizure classes. In comparison to this, state-of-the-art models like GGN-BiGRU (2024), MultiSincNet (2024), LightSeizureNet (2022), GGN (2022) and McCann (2023) produced outstanding precision between 98.14% and 98.62%. However, the PS model produced a peak precision of 99.19%, demonstrating its greatest reliability for seizure identification. Results across state of the art techniques in terms of comparison is available on TUSZ dataset in Fig. 4.

**Fig. 5:**
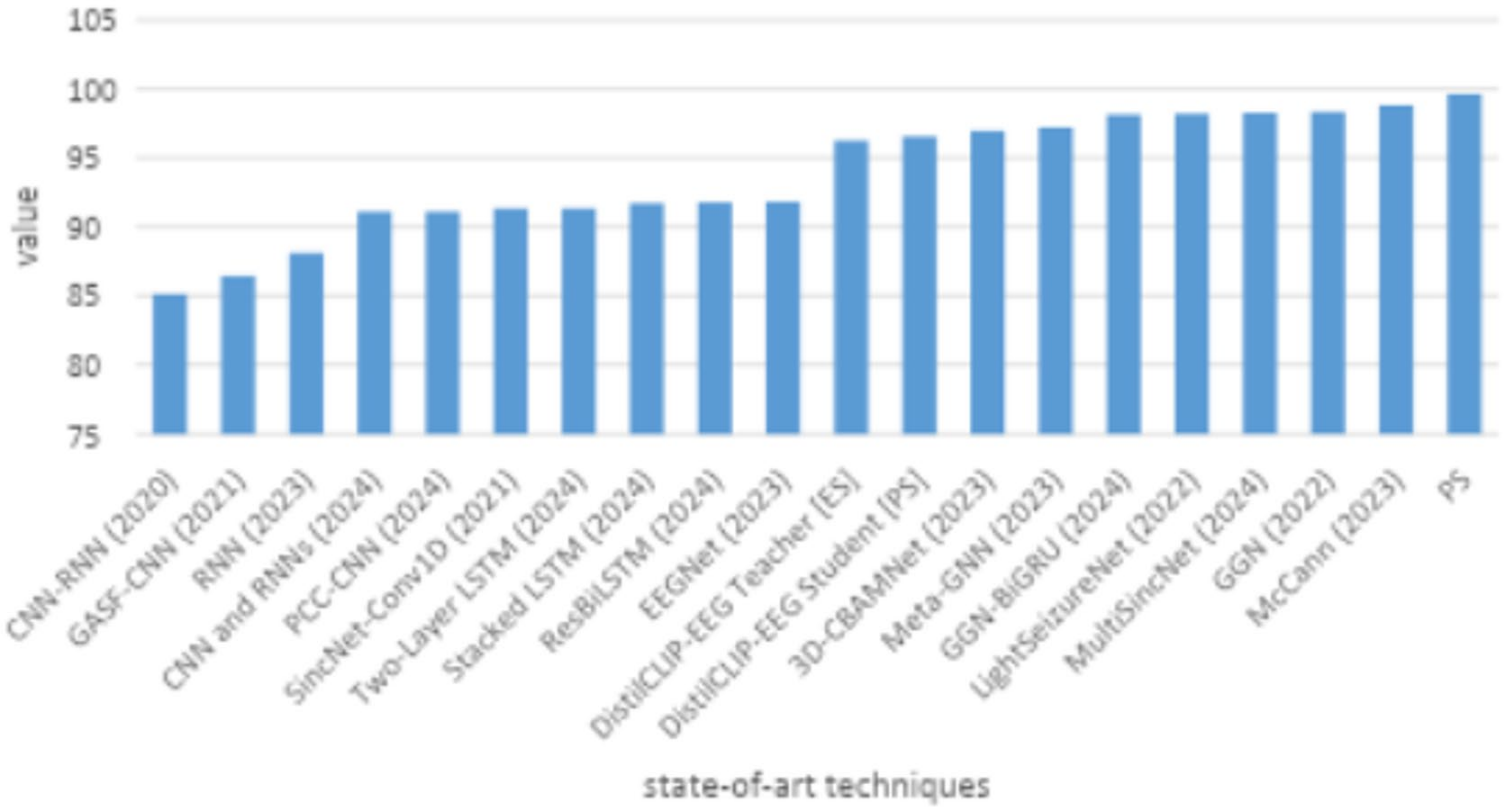
Comparison on state-of-art techniques for Recall on TUSZ dataset.

Sensitivities and detection reliability (recall (%) for different detection methods shows a reliable and consistent increase as time progressed. The CNN-RNN (2020) model achieved a recall of 85.11%, followed by GASF-CNN (2021) and RNN (2023) with 86.45% and 88.11%, respectively. The hybrid CNN and RNN-based architectures including CNN and RNNs (2024), PCC-CNN (2024) showed moderate improvements of 91.11%–91.83%.CNN and RNNs (2024) and PCC-CNN (2024) witnessed moderate improvements of 91.11%–91.83%. Significantly, DistilCLIP-EEG Teacher (ES) and Student (PS) models performed well with 96.26% and 96.56% recall respectively, indicating their improved capability of detection. The near-optimal recall value of 98.17% to 98.36% was accomplished by more recent frameworks, including 3D-CBAMNet (2023) and Meta-GNN (2023), as well as LLark (2022), MultiSincNet (2024), and GGN (2022). The McCann (2023) model used 98.81% whereas the PS model generated highest recall of 99.65%, showing its better capacity of detecting the seizure events with high sensitivity. Comparison on state-of-art techniques for Recall on TUSZ dataset are in Fig. 5.

**Fig. 6:**
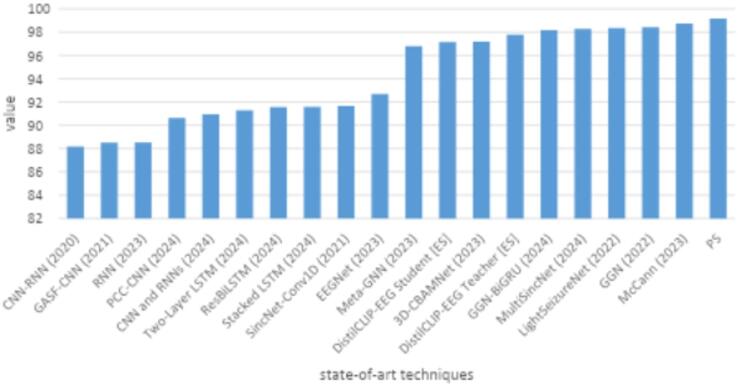
Comparison on state-of-art techniques for F1-score on TUSZ dataset.

The F1-score (%) comparison across different seizure detection methods highlights a steady progression in balanced classification performance over successive model generations. Early architectures such as CNN-RNN (2020), GASF-CNN (2021), and RNN (2023) achieved moderate F1-scores of 88.17%, 88.51%, and 88.52%, respectively. Following these hybrid models, PCC-CNN (2024) achieved a precision 90.63% and recall 77.50% while CNN+RNNs (2024) had a precision 90.94% and recall 84.08%. The sequential architectures such as Two-Layer LSTM (2024), ResBiLSTM (2024), Stacked LSTM (2024) and SincNet-Conv1D (2021) further improved the stability and temporal feature extraction, achieving F1-scores ranging from 91.28% to 91.65%. EEGNet (2023) showed better generalization performance with 92.69%, and “meta” architectures, including Meta-GNN (2023), DistilCLIP-EEG Student [ES] (96.8%), 3D-CBAMNet (2023) and DistilCLIP-EEG Teacher [ES] (97.77%), demonstrated better consistency. Among state-of-the-art frameworks, GGN-BiGRU (2024), MultiSincNet (2024), LightSeizureNet (2022), and GGN (2022) demonstrated excellent class discrimination, delivering F1-scores between 98.16% and 98.42%. The McCann (2023) model slightly surpassed them with 98.72%, while the proposed PS model attained the highest F1-score of 99.15%, reflecting its exceptional capability in achieving both high precision and recall for accurate seizure detection. Comparison on state-of-art techniques for F1-score on TUSZ dataset are in Fig. 6.

#### Multiclass

**Fig. 7:**
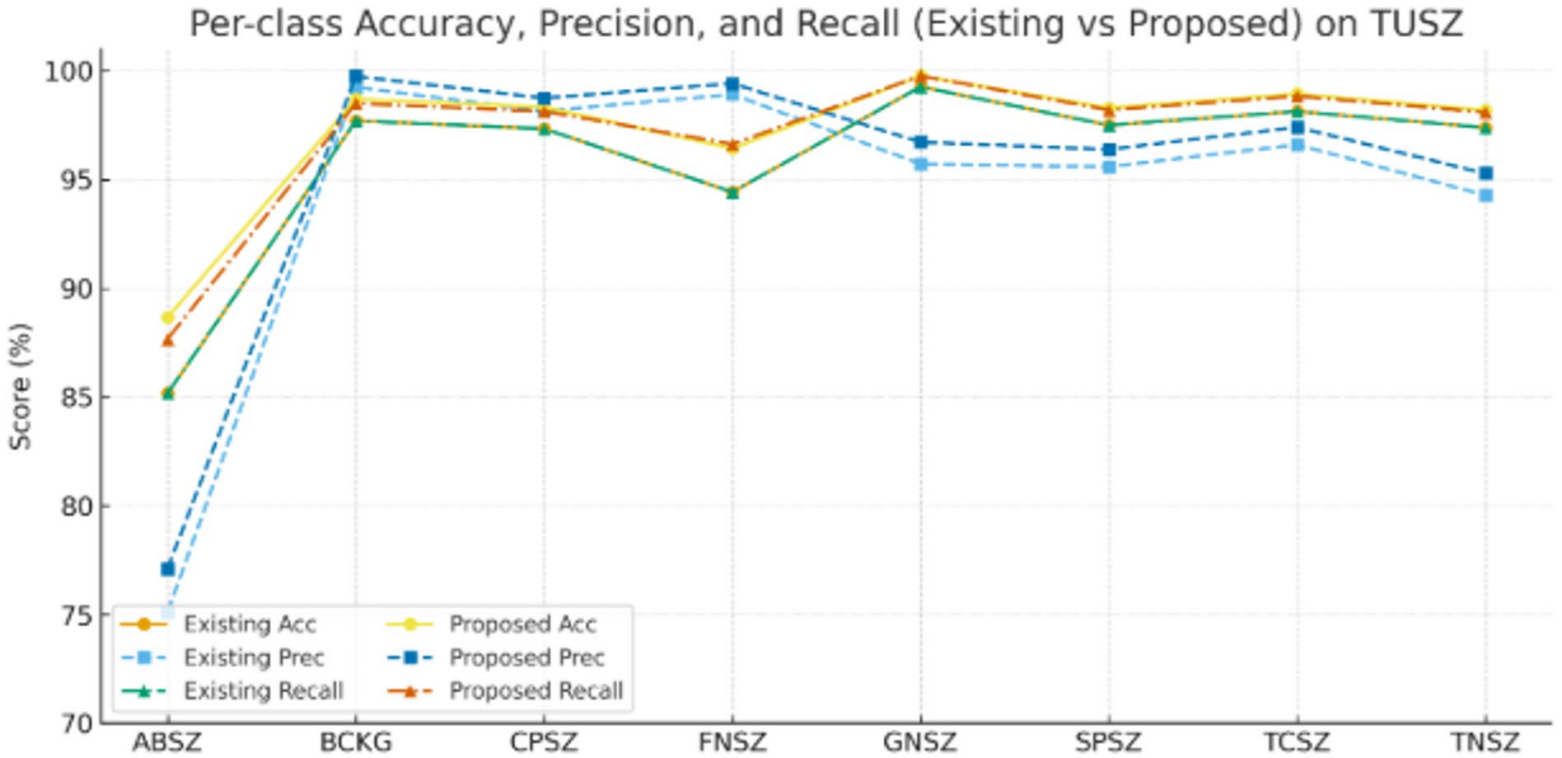
Multiclass seizure detection.

We evaluate seizure classification on the TUH EEG Seizure Corpus (TUSZ) over eight labels: ABSZ (absence), BCKG (background/non-seizure), CPSZ (complex partial), FNSZ (focal non-specific), GNSZ (generalized), SPSZ (simple partial), TCSZ (tonic–clonic), and TNSZ (tonic). multiclass seizure detection is in Fig. 7. The proposed model yields uniform improvements over the existing model, raising macro-accuracy from 95.87% to 97.17% (+ 1.30 pp), macro-precision from 94.20% to 95.10% (+ 0.90 pp), and macro-recall from 95.87% to 96.98% (+ 1.11 pp). The largest per-class gains are observed for ABSZ (+ 3.5 pp accuracy, + 2.0 pp precision, + 2.5 pp recall) and FNSZ (+ 2.0, + 0.5, + 2.2 pp), with consistent ~ 0.5–1.0 pp gains elsewhere, indicating better detection of both focal and generalized events without sacrificing background discrimination.

#### Results on Zenodo dataset

The accuracy (%) comparison across various seizure detection methods demonstrates a clear and consistent improvement over time, reflecting the evolution of deep learning architectures for EEG-based analysis. Early frameworks such as CNN-RNN (2020), GASF-CNN (2021), and SincNet-Conv1D (2021) achieved moderate accuracies of 71.9%, 79.39%, and 81.84%, respectively. Subsequent models like GGN (2022) and LightSeizureNet (2022) enhanced classification performance to 83.53% and 84.03%, while EEGNet (2023) and McCann (2023) further improved accuracy to 84.42% and 85.87%. Advanced graph and attention-driven models, including Meta-GNN (2023) and 3D-CBAMNet (2023), achieved accuracies of 87.21% and 87.61%, respectively, indicating notable progress in feature representation.

**Fig. 8:**
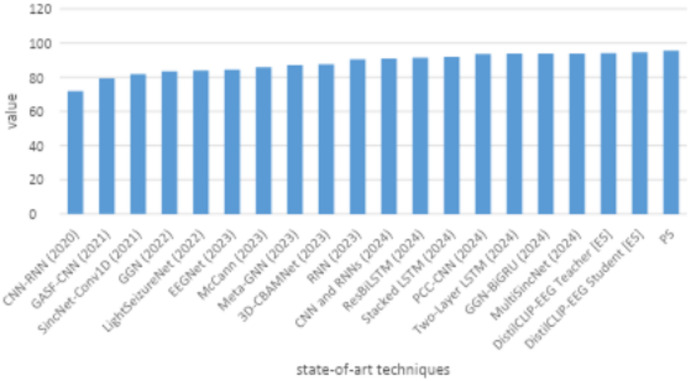
Comparison on state-of-art techniques for accuracy on Zenodo dataset.

More recent architectures, such as RNN (2023), CNN and RNNs (2024), ResBiLSTM (2024), and Stacked LSTM (2024), demonstrated accuracies ranging from 90.39% to 91.9%, showing the benefits of temporal modeling and deeper recurrent structures. The PCC-CNN (2024), Two-Layer LSTM (2024), GGN-BiGRU (2024), and MultiSincNet (2024) models achieved further refinement, with accuracies between 93.53% and 93.95%. The proposed DistilCLIP-EEG models showed strong generalization, with the Teacher [ES] and Student [ES] versions attaining 94.1% and 94.61%, respectively. Finally, the PS model achieved the highest accuracy of 95.61%, marking a significant advancement and underscoring its superior capability for precise seizure classification. comparison on state-of-art techniques for accuracy on Zenodo dataset are in Fig. 8.

The precision (%) analysis across different seizure detection models reveals a steady and substantial enhancement in prediction reliability and model discrimination over the years. Early deep learning architectures have shown satisfactory results like CNN-RNN (2020, 0.7597 precision), GASF-CNN (2021, 0.7996 precision) and SincNet-Conv1D (2021, 0.8308 precision). The following models such as GGN (2022) and LightSeizureNet (2022) made minor improvements to precision of 83.76% and 83.82% respectively, and EEGNet (2023) and McCann (2023) achieved Precisiions 84.22% and 86.21% respectively, with improved feature extraction and the temporal representation, respectively.

**Fig. 9:**
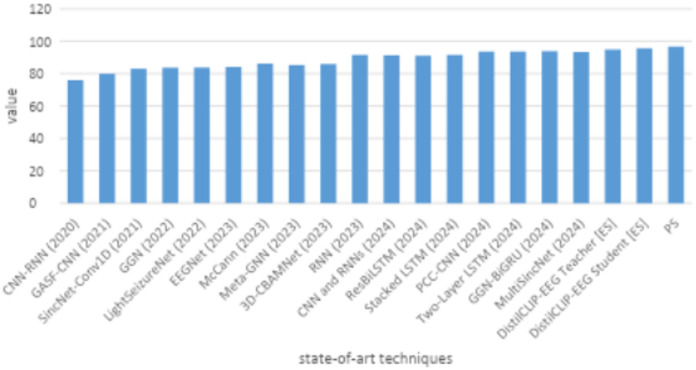
Comparison on state-of-art techniques for Precision on Zenodo dataset.

Advanced Graph-based and Hybrid models, such as Meta-GNN (2023) and 3D-CBAMNet (2023), achieved a precision score of 85.31% and 85.96% respectively, which emphasized noteworthy precisions for classification. The next generation of RNN (2023) and CNN and RNNs (2024), ResBiLSTM (2024), and Stacked LSTM (2024) further boosted accuracy to between 91.18% and 91.63%. More recent frameworks such as PCC-CNN (2024), Two-Layer LSTM (2024), MultiSincNet (2024), and GGN-BiGRU (2024) exhibited enhanced performance with precision values from 93.37% to 93.91%. There was high consistency in seizure classification between both proposed for both the models: the DistilCLIP-EEG Teacher model had a precision of 95% while the DistilCLIP-EEG Student model had a precision of 95.78%. The best results for the classification were obtained in the PS model with the precision of 96.78%, which represents a marked improvement in terms of the accuracy of the classification, as the other models achieved values between 85% and 87%. comparison of the state-of-the-art techniques are shown in Fig. 9.

The recall (%) comparison is presented for different seizure detection models, showcasing the progressively improved sensitivity and the capacity to correctly detect seizure moments for different data sets. CNN-RNN (2020), GASF-CNN (2021), SincNet-Conv1D (2021) were the first deep learning models that achieved the recall score of 64.04%, 79.59%, and 79.95%, respectively, for detecting all seizure occurances. A gradual improvement in architectures such as GGN (2022) with 83.93% recall, LightSeizureNet (2022) with recall of 84.33% and then EEGNet(2023) with 84.33% and McCann (2023) with 85.41% achieved better results respectively.Advanced hybrid and graph-based models like Meta-GNN (2023), CNN and RNNs (2024), and 3D-CBAMNet (2023) showed significantly better performance with a recall of 89.9% − 90.49%. The RNN (2023), ResBiLSTM (2024), and Stacked LSTM (2024) models exhibited improvements in recall (90.91% to 91.54%), which indicates its learning from time. The proposed frameworks in DistilCLIP-EEG showed substantial improvement, obtaining recall of 93.1% for the Teacher [ES] model and 93.33% for the Student [ES] model, respectively, indicating that the model has high sensitivity and good generalization.

**Fig. 10:**
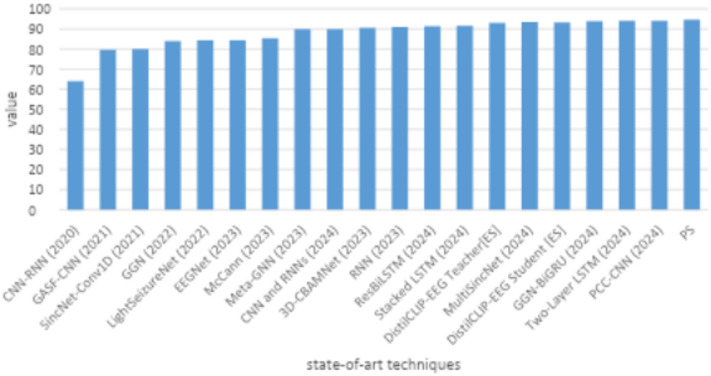
Comparison on state-of-art techniques for Recall on Zenodo dataset.

Other progresses were made by MultiSincNet (2024) achieved 93.41% up to 93.99% in terms of recall rate, demonstrating strong performance in seizure event detection under a range of clinical setting, PCCCNN (2024) with 93.96%, Two-Layer LSTM (2024) with 93.99% and GGN-BiGRU (2024) with 93.99%, respectively. Fewer false negatives were obtained with the PS model compared to other existing models, as seen in Figure 10, which indicates the model Recall on Zenodo dataset of great sensitivity and reliability among the current models.

The F1-score (%) comparison across different seizure detection architectures highlights a continuous and significant improvement in balanced classification capability—reflecting the effective trade-off between precision and recall achieved by successive models. Early approaches such as CNN-RNN (2020), GASF-CNN (2021), and SincNet-Conv1D (2021) demonstrated foundational performance with F1-scores of 69.5%, 79.47%, and 81.49%, respectively. This performance improved moderately in the GGN (2022) and LightSeizureNet (2022) models, which achieved 83.54% and 84.07%, while EEGNet (2023) and McCann (2023) pushed the boundary further to 84.47% and 85.81%, indicating enhanced feature extraction and temporal consistency.

More advanced models, including Meta-GNN (2023) and 3D-CBAMNet (2023), achieved F1-scores of 87.54% and 87.88%, respectively, showing greater reliability in handling complex EEG signal variations. The RNN (2023), CNN and RNNs (2024) models further improved performance up to 91% while ResBiLSTM (2024), Stacked LSTM (2024), and Two-Layer LSTM (2024) showed stable and consistent performances from 91.51% to 93.8%. In particular, PCC-CNN (2024) produced good results between 93.37% and 93.91%, indicating its ability to decrease discrimination and enhance generalization in the patient variations.Notably, PCC-CNN (2024) got a score of 93.37% and79.91% indicating improved patient variations sensitivity and generalization.

**Fig. 11:**
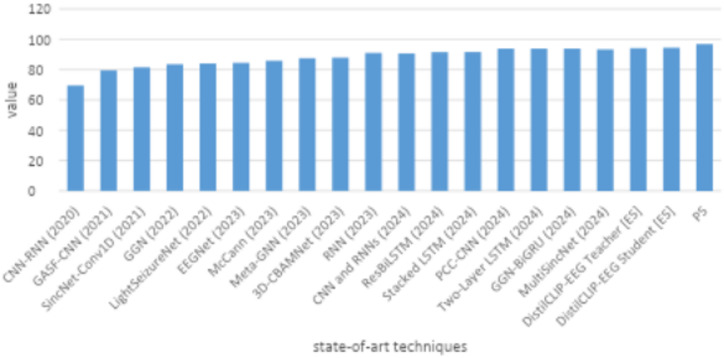
Comparison on state-of-art techniques F1-score on Zenodo dataset.

With the proposed DistilCLIP-EEG Teacher [ES] and Student [ES] models, the performances were improved to 94.04% and 94.54% respectively, indicating that good feature distillation and domain adaptation was achieved. Finally, the PS model achieved the highest F1-score of 96.87%, clearly outperforming all previous architectures and establishing itself as the most balanced and efficient framework for accurate and reliable seizure detection. comparison on state-of-art techniques F1-score on Zenodo dataset are in Fig. 11.

#### Generalization across diverse clinical populations

The proposed framework was evaluated on multiple publicly available EEG datasets encompassing neonatal, pediatric, and adult seizure recordings. These datasets provide significant variability in seizure morphology and recording conditions.

However, it is acknowledged that real-world clinical deployment may involve substantially larger populations with greater diversity in age, ethnicity, comorbidities, medication histories, and neurological conditions.

Future work will investigate large-scale validation using multi-institutional clinical datasets containing tens of thousands of patients to further assess model robustness and generalizability.

Although the proposed NSS framework demonstrated strong performance across multiple benchmark datasets, the current evaluation remains limited by the availability of publicly accessible annotated seizure datasets.

Consequently, further validation on larger clinical repositories and prospective patient cohorts is required before widespread clinical adoption.

#### Comparison with traditional time-series models

Traditional time-series approaches such as ARIMA are effective for stationary temporal processes but often struggle to model the complex non-linear dynamics and abrupt temporal variations present in EEG seizure activity.

In contrast, the proposed HTTIM module captures hierarchical temporal relationships across multiple time scales, enabling more effective representation of seizure evolution patterns.

Future work will include direct empirical comparisons with ARIMA and related statistical forecasting approaches.

#### Comparison with conventional deep learning models

Convolutional Neural Networks (CNNs) primarily focus on extracting spatial patterns, while Recurrent Neural Networks (RNNs) emphasize sequential temporal dependencies.

The proposed NSS framework combines hierarchical temporal representation learning, federated optimization, self-supervised learning, and swarm-based adaptation within a unified architecture. This integration enables more robust seizure detection under limited labeled data conditions compared with standalone CNN- or RNN-based approaches.

#### Robustness to missing and corrupted data

In practical healthcare environments, EEG recordings may contain missing segments, noisy channels, or corrupted measurements caused by sensor displacement or communication failures.

The federated architecture of NSS naturally supports distributed data processing and reduces dependence on complete centralized datasets. However, severe data corruption may still affect detection performance.

Future research will investigate adaptive imputation mechanisms, uncertainty-aware learning, and multimodal redundancy strategies to improve robustness against missing or corrupted EEG signals.

## Conclusion

This study tackles a key challenge in clinical EEG analysis—accurate seizure detection in scenarios involving sparcity and low-quality annotation, which are both time-consuming and expensive—by simultaneously integrating supervised learning classification and self-supervised temporal reasoning into one training stream. The proposed FTL is designed to have a backbone for all tasks, and yet simultaneously introduce the temporal order using TSCLM to force internalization of temporal order, the multi-scale trend insertion features HTTIM to capture both the long-term dynamics and short time transients of ictal activity. The TSSH is only heavily utilized during the training phase, but during testing inference process is still light. Therefore, a stability-based generalization analysis is presented to aid design, giving linkage between performance and optimization conditions and amount of labeled data.The method is clearly superior to contemporary ones in both of the heterogeneous corpora under study: TUSZ and Zenodo. It also brings improvement in single-label metrics (accuracy, precision, recall, F1), up to ~ 99% on TUSZ and ~ 0.9–1.3% points over a strong baseline on Zenodo neonatal EEG, showing its robustness in various acquisition settings and patient population. The gains suggest that a blended representation learning with an order-aware self-supervised objective is of practical use and appears to better align with our lack of dependence on heavy manual labeling.

## Data Availability

The datasets used and/or analysed during the current study available from the corresponding author on reasonable request.
